# Efficiency of Identification of Blackcurrant Powders Using Classifier Ensembles

**DOI:** 10.3390/foods13050697

**Published:** 2024-02-24

**Authors:** Krzysztof Przybył, Katarzyna Walkowiak, Przemysław Łukasz Kowalczewski

**Affiliations:** 1 Department of Dairy and Process Engineering, Faculty Food Sciences and Nutrition, Poznań University of Life Sciences, 31 Wojska Polskiego St., 60-624 Poznań, Poland; 2 Department of Physics and Biophysics, Faculty Food Sciences and Nutrition, Poznań University of Life Sciences, 28 Wojska Polskiego St., 60-637 Poznań, Poland; katarzyna.walkowiak@up.poznan.pl; 3 Department of Food Technology of Plant Origin, Faculty Food Sciences and Nutrition, Poznań University of Life Sciences, 31 Wojska Polskiego St., 60-624 Poznań, Poland; przemyslaw.kowalczewski@up.poznan.pl

**Keywords:** machine learning, classifiers ensembles, metaclassifier, random forest (RF), gray-level co-occurrence matrix (GLCM), texture, blackcurrant powders

## Abstract

In the modern times of technological development, it is important to select adequate methods to support various food and industrial problems, including innovative techniques with the help of artificial intelligence (AI). Effective analysis and the speed of algorithm implementation are key points in assessing the quality of food products. Non-invasive solutions are being sought to achieve high accuracy in the classification and evaluation of various food products. This paper presents various machine learning algorithm architectures to evaluate the efficiency of identifying blackcurrant powders (i.e., blackcurrant concentrate with a density of 67 °Brix and a color coefficient of 2.352 (E520/E420) in combination with the selected carrier) based on information encoded in microscopic images acquired via scanning electron microscopy (SEM). Recognition of blackcurrant powders was performed using texture feature extraction from images aided by the gray-level co-occurrence matrix (GLCM). It was evaluated for quality using individual single classifiers and a metaclassifier based on metrics such as accuracy, precision, recall, and F1-score. The research showed that the metaclassifier, as well as a single random forest (RF) classifier most effectively identified blackcurrant powders based on image texture features. This indicates that ensembles of classifiers in machine learning is an alternative approach to demonstrate better performance than the existing traditional solutions with single neural models. In the future, such solutions could be an important tool to support the assessment of the quality of food products in real time. Moreover, ensembles of classifiers can be used for faster analysis to determine the selection of an adequate machine learning algorithm for a given problem.

## 1. Introduction

Along with the development of technology, the view of food quality and safety has changed. The processes of food production, management, distribution, and consumption have evolved significantly over the past few years. These developments resulted from a greater focus on strengthening food quality and safety control systems [1,2,3,4]. When looking at recent developments in climate change [5], consumer behavior and preferences [6], and food adulteration problems, it becomes a challenge to ensure food safety for the benefit of human health [7]. The successive introduction of more modern solutions in the food industry aimed at improving local food quality control systems affects the improvement of global food safety [8,9]. As a result of continued technological advances, especially in the field of artificial intelligence, food quality control seems likely to intensify [9]. These changes in both quality and food safety could become an important element in achieving global economic success.

The aforementioned artificial intelligence (AI) can make a significant contribution to improving the quality as well as safety of food through, among other things, the online monitoring of food quality at each stage of the process [10]. AI can manage food storage collections, using sorting, packaging, and cleaning processes to maintain optimal storage conditions [11,12]. AI will work to increase the efficiency of sourcing raw materials by, among other things, optimizing parameters in the process, which will help reduce food waste. The idea of repeatable processes to obtain homogeneous food products for quality uniformity is also increasingly possible with the help of product classification [13], contamination detection [14], and defects, as well as quality assessment of these foods [15,16,17].

In view of the above, the search for effective methods to develop products ensuring quality and safety is crucial given the protection of consumer health. Many traditional analytical methods in food are expensive and time-consuming, e.g., nuclear magnetic resonance (NMR) or Fourier-transform infrared (FTIR) [18]. Analytical methods are also used to identify foods based on various attributes including components or organic compounds in a research sample. However, the classification of food products often requires the use of further analytical methods. This contributes to the need for comparative methods that can classify a food product just as quickly and effectively. Machine learning, among others, through image classification, and extracting features from an image, is becoming an alternative tool when evaluating food quality and safety. Therefore, it seems reasonable to look for innovative techniques to ensure fast yet effective food inspection, while minimizing financial and energy expenditure.

Machine learning using algorithms has provided an innovative approach to effectively analyze multidimensional data. Considering the analysis of raw materials and food products, machine learning algorithms can effectively identify important attributes (parameters) of food that can affect its quality [19,20,21,22,23]. An interesting tool is ensembles of classifiers due to their optimal estimation of data as a result of product classification [24,25,26]. Currently, there are efforts to make data recognition more effective and efficient using artificial intelligence methods. Classifier ensembles represent one method of machine learning in which multiple algorithms are combined to result in better performance than individual models. As a result of tuning hyperparameters for individual models, it is possible to identify these data efficiently, even when they are highly complex. This, in turn, translates into improved model generalization capabilities. When looking at the context discussed by the team of Liu et al., 2021 [27], that machine learning algorithms can have poor efficiency due to attribute selection, it also seems reasonable to tune hyperparameters in models [28].

This study focused on identifying currant powders obtained via the low-temperature drying process due to the different types of carriers. Blackcurrant powders as food products are rich in vitamins, minerals, and antioxidants [29]. They contain anthocyanins, which belong to a group of natural colorants of plant origin [30,31]. Anthocyanins are powerful antioxidants that contribute to, among other things, reducing the risk of cardiovascular disease and also controlling blood sugar levels to counter diabetes [30]. A major advantage of fruit powders is their sustainability during storage. It is due to the method of obtaining powders associated with the reduction in, among other things, water content, which in turn translates into the retention of a large amount of fruit nutrients for a longer period of time [32]. For food, the way soft fruits are transported is also a difficulty, leading to their wastage due to sudden spoilage. While recognizing the problem of sourcing food products only seasonally, fruit powders seem to be an alternative solution.

The utilitarian aim was to develop an efficient way to recognize classes of currant powders using advanced machine learning algorithms. We applied artificial intelligence methods using ensembles of classifiers. Considering the authenticity and maintenance of quality as a result of sourcing these food products, the process of identifying different classes of currant powders also becomes important due to their physical and chemical properties [32]. This attempt to apply machine learning algorithms will allow efficient (authentic) recognition of properties between different types of currant powders. The different machine learning techniques were tested, obtaining efficient yet optimal comparisons. The proposed solution based on using ensemble methods of machine learning in a single metaclassifier [33,34,35] to control the quality assessment of food products increases the efficiency of model generalization compared to each of the component algorithms. Machine learning can contribute to improving quality standards, and controlling adulteration, as well as the efficiency of obtaining final products, i.e., blackcurrant powders.

## 2. Materials and Methods

### 2.1. Sample Collection

The research material consisted of blackcurrant powders obtained through low-temperature drying. The drying stage of the currant solution process was controlled at a fixed inlet air temperature of 80 °C and an outlet air temperature of 50 °C to maintain the properties of the nutrients [13]. The currant solution was the obtained currant concentrate and an appropriate amount of the selected carrier. In the process of realizing the project, concentrated blackcurrant juice (blackcurrant concentrate) with a density of 67 °Brix and a color coefficient of 2.352 (E520/E420) was obtained in the amount of 5 kg from a batch of 250 kg of this product from the company Białuty Sp. z o.o. More details on how to obtain the solution and the properties of the currant concentrate, as well as the carriers, are included in the research study by Przybyl et al., 2023 [29]. As part of the spray drying experiments, it was determined that the recognition of fruit powders would take place for the currant solution, which contains only 30% of the carrier. In order to obtain blackcurrant powders with a carrier, those that are indeed common in food were selected, i.e., cellulose (C70), inulin (IN70), maltodextrin (MD70), whey milk Protein (W70), fiber (F70) and gum Arabic (G70) [29].

### 2.2. Data Collection

In the first step of the research work, a data set was prepared based on microscopic images (scanning electron microscopy) of blackcurrant powders. The procedure for the preparation of the microscopic image was based on the information prepared when taking microscopic images involving the raspberry powders Przybyl, 2021 [13]. The digital images with a resolution of 2048 × 1576 in .TIFF format of currant powder microparticles were obtained at 500 times magnification at a scale of 100 um (a total of 630 digital images). The dataset (learning set) contained 6 different classes of currant powders. Every class represents the obtained currant powder as a result of low-temperature drying with a specific carrier, and moreover, the same number of images, i.e., 105 microscopic images each in the catalog. The learning set was assigned 6 catalogs corresponding to blackcurrant powders with the selected carrier ID, i.e., class_W70, class_MD70, class_IN70, class_G70, class_F70, and class_C70.

### 2.3. Feature Extraction

In the next step, the preprocessing of the learning set was performed. Each 2048 × 1576 digital image with a .TIFF extension (primary image) was transformed into an 8-bit 1400 × 1400 secondary image with a .jpg extension. The batch image transformation was performed with the help of the developed proprietary algorithm and the included free Python Imaging Library (PIL) in Python ver. 3.9. PIL is an image processing tool in Python language. In the next step, an image segmentation algorithm was developed to acquire an image resolution of 1400 × 1400. Also, so-called image cropping was performed with a certain step in relation to the parameters of the x- and y-axes, without distorting the image. The final step required transforming the images pointwise by 90, 180, and 270 degrees. These steps yielded 105 8-bit images with a resolution of 1400 × 1400 with .jpg extension for each class in the learning set.

The test collection contained 210 images (6 classes of 35 digital currant powder images each). In the test set, in accordance with the principle of machine learning, those images that belonged to the learning set were not included. The image processing procedure was carried out similarly to the learning set with the exclusion of point transformation. Datasets in the learning (class_train) and test (class_test) sets, i.e., actual images of blackcurrant powders in the morphological structure, required reading and then the performance of a feature extraction process. In order to carry this out this process, the Canny filter was used. This is the most commonly used operator for revealing edges from an image. This process made it possible to detect microparticles of currant powders with a given media type in the 8-bit secondary image.

### 2.4. Texture Analysis

In the next step, an algorithm was developed to go from an image to a data vector. The aim of the activity was to prepare the image as a data vector for image analysis using representative features. In this step, the image texture for blackcurrant powder recognition was applied by using the well-known gray-level co-occurrence matrix (GLCM) [36], and the features energy, entropy, correlation, dissimilarity, homogeneity, and contrast were determined from the image for each case in the learning set [37,38,39,40]. The GLCM features allow for the description of the texture of a selected part of the image with several numerical values. Using the GLCM matrix, the relationship between the pixel space and the gray level of the matrix can be described, allowing the texture features to be determined and calculating how often a pair of pixels with certain values occur over the gray band of the image (Przybyl et al., 2018) [36]. The GLCM effectively extracts pixel fragments step by step to analyze the frequency of pixel pairs over the gray band of an image. Methods for calculating GLCM features were proposed in the work of Haralick et al. 1973 [41,42,43]. Their implementation in the analysis of an image as a bitmap is possible, among others, using the Python language (Python ver. 3.9) and the sci-kit image library. Individual GLCM discriminants are calculated according to the following formulas [14,42,43,44]:

(1) $$\mathrm{Contrast}:\;\sum P_{i,j}{\left(i-j\right)}^{2},$$

(2) $$\mathrm{Dissimilarity}:\;\sum P_{i,j}\left|i-j\right|,$$

(3) $$\mathrm{Homogeneity}:\;\sum \frac{P_{i,j}}{1+{\left(i-j\right)}^{2}},$$

(4) $$\mathrm{Energy}:\;\sqrt{\sum P_{i,j}^{2}},$$

(5) $$\mathrm{Correlation}:\;\sum P_{i,j}\left[\frac{\left(i-\mu_{i}\right)\left(j-\mu_{j}\right)}{\sqrt{\left(\sigma_{i}^{2}\right)\left(\sigma_{j}^{2}\right)}}\right],$$

(6) $$\mathrm{Entropy}:\;\sum p_{i,j}{log}_{2}p_{i,j}$$

### 2.5. Classifier Ensembles

In the next phase of the research problem, 35 classifiers were used to identify blackcurrant powders. In machine learning, different classifiers were tuned due to their hyperparameters [45,46,47] in order to find the most effective model for identifying currant powders. One of the 35 classifiers was a metaclassifier, which was used to improve the accuracy of classified data for comparison with single classifiers. The single machine learning algorithms used were decision trees (DF), random forest (RF), adaptive boosting (AdaBoost), bootstrap aggregating (bagging), stochastic gradient descent classifier (SGDClassifier), RidgeClassifier, perceptron, multilayer perceptron (MLPPerceptron), and GaussianProcessClassfier. DFs partition data into subgroups using specific decision conditions (hyperparameters) [48]. RF is one of the machine learning methods that uses multiple decision trees for classification [49,50,51]. The benefit of RF with respect to DF, seems to be the matching of each tree in RF to a different set of data, causing an increase in classification efficiency and thus controlling over-fitting of this model for the data. AdaBoost has focused on adaptive error matching of classifiers, increasing precision, especially for those weak classifiers [52,53,54]. The bagging algorithm can quickly generate multiple copies of the same classifier while learning different subsets of data and generalizing the model [55,56]. SGDClassifier applies a gradient optimization technique, minimizing loss functions [57]. It can be a very flexible yet effective model, especially in the classification aspect. Another algorithm is RidgeClassifier based on ridge regression [58]. This model was characterized by a high need for data control in optimizing the results, among other things. Another algorithm that was used to identify powders is GausianProcessClassfier [59]. GausianProcessClassfier is a probabilistic type model used to estimate the probability distribution of data in a set. The model can be characterized by the fact that it offers flexibility in adapting to the learning data, especially when the data are irregular or complex due to their case complexity. Recent algorithms are perceptron- and MLPClassifier-type models [3,60,61,62]. These are classifiers based on the perceptron structure, which consists of 3 layers (input, hidden, and output) and gains from the greatest tuning of hyperparameters.

As a result, the set of classifiers for currant powder recognition consisted of 34 individual different classifiers, which differed, among other things, in the type of algorithm and choice of hyperparameters when initializing each model, and 1 metaclassifier. Table 1 presents the structure of each of these classifiers.

Table 1 defines the hyperparameter tuning steps for each algorithm. Hyperparameters in MLPClassifier neural networks specify input variables such as the number of hidden layers, the number of neurons in each hidden layer, the activation function, the optimization algorithm, and the loss function. In the case of applying hyperparameters to decision trees, the key element in the structure was the max-depth hyperparameter, which denoted the max-depth, i.e., the number of levels into which the tree can branch [63]. This is the main hyperparameter, among others, affecting the optimization of the model to avoid so-called overfitting [64]. In random forest, among the many hyperparameters, those that affect both the evaluation of the quality of the model and the control against overfitting of the algorithm were selected. As in decision trees, the level of branching was determined using the max-depth hyperparameter. In addition, the hyperparameters n_esitmators responsible for improving the model’s classification accuracy and criterion = gini were included to assess measures of the model’s quality [65,66]. In the case of SGDClassifier, an additional hyperparameter affecting the optimization of the algorithm was the penalty parameter. The penalty hyperparameter assisted in the process of model tuning (optimization during learning) refers to the type of penalty set on erroneously specified cases in the test set [67]. The purpose of regularization is to prevent model overfitting. The max-iter hyperparameter indicates at which epoch the model learning process should end [68]. The perceptron-type algorithm uses the tol = 1 × 10^−3^ hyperparameter, which is responsible for the tolerance to the error (tol) made on the test set. The smaller the tol value, the greater the chance of obtaining a more effective model accuracy result. The last algorithm in the set of classifiers is MetaClassifier. MetaClassifier is a combination of 3 individual classifiers such as logistic regression, bagging, and random forest.

### 2.6. Model Training and Testing

In this phase, the division of the data into a learning set and a test set was carried out. The division of the learning set to the test set was carried out in a ratio of 75:25. The choice of learning method, e.g., training and testing, translates into the fact that the data are divided into two sets of training and testing in a ratio of 75% to 25%. In Python, according to the specifics of the issue, it is performed using the train_test_test method, the so-called split sets. In the training and testing method, the process of selecting data randomly is carried out, i.e., the indexes of the learning cases are shuffled in a random order in order not to affect specific learning cases (i.e., with a given index) when learning models. It is important that data partitioning is carried out via normalization or standardization so as not to obtain bad model quality metrics (model performance). In this work using Python, features were normalized using the StandardScaler tool. In the next step, the process of teaching ensembles of classifiers was carried out. As part of the activity, a metaclassifier was added to compare the effectiveness of recognizing currant powders.

As part of the validation of the test set, learning quality indicators were presented by accuracy, precision, recall, and F1-score. A confusion matrix was used for the prediction of the acquired data. The learning process was performed using Python ver 3.9.

## 3. Results and Discussion

**Figure 1:**
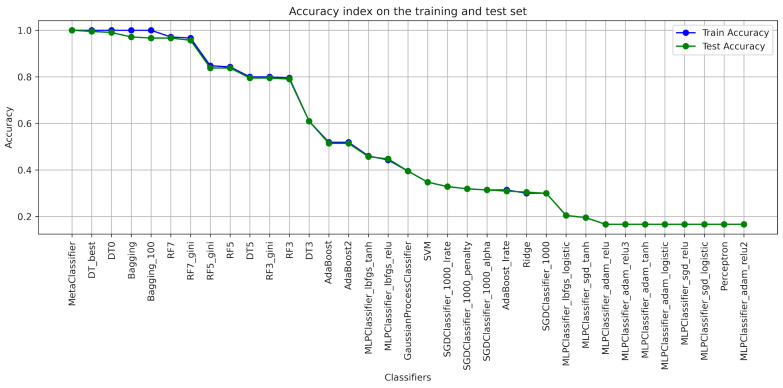
Classifier accuracy rate on the test and learning set for the individual GLCM attributes—ENTROPY.

**Figure 2:**
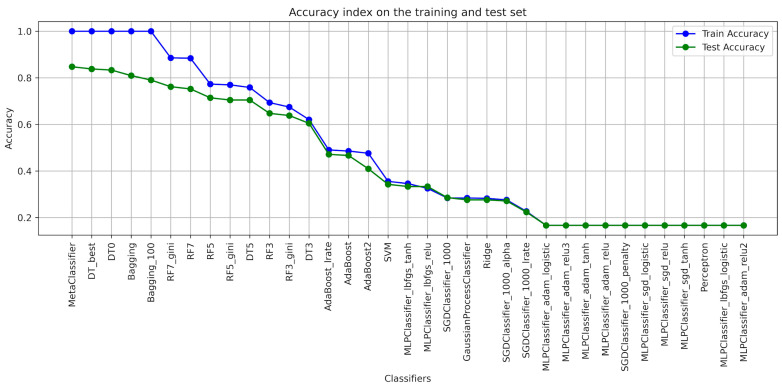
Classifier accuracy rate on the test and learning set for the individual GLCM attributes—ENERGY.

**Figure 3:**
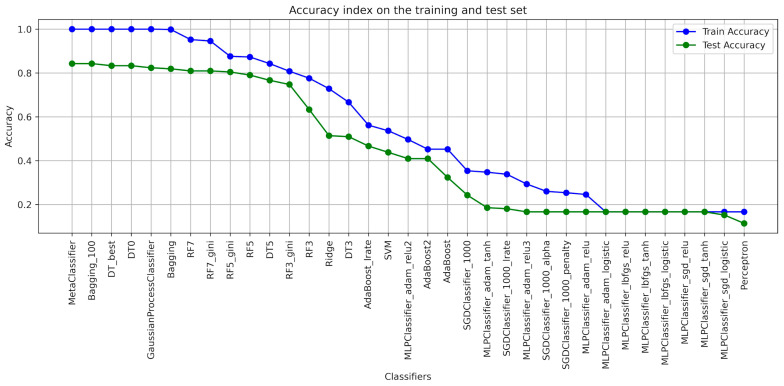
Classifier accuracy rate on the test and learning set for individual GLCM attributes—CONTRAST.

**Figure 4:**
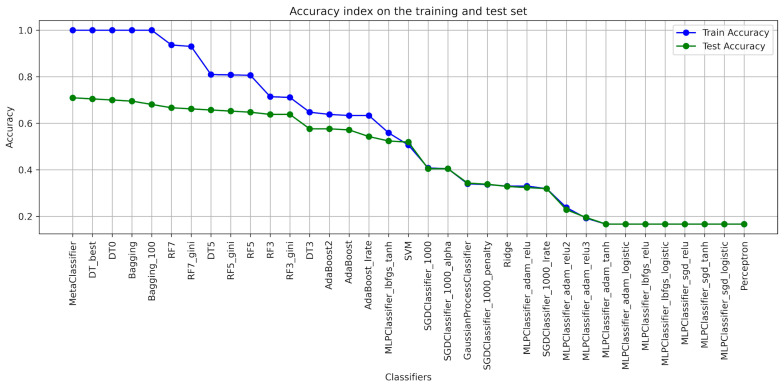
Classifier accuracy rate on the test and learning set for the individual GLCM attributes—COOREALTION.

**Figure 5:**
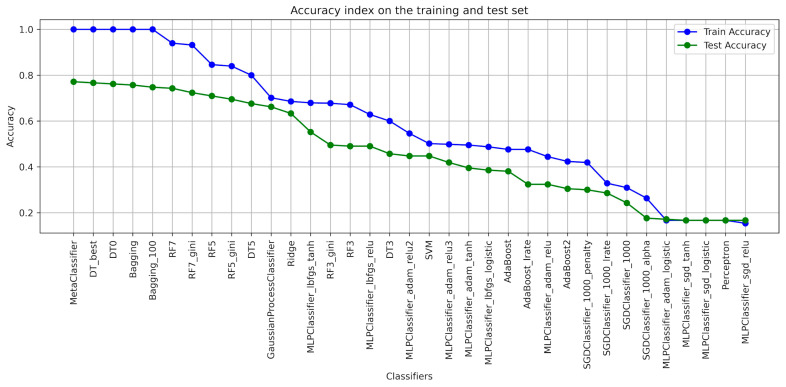
Classifier accuracy rate on the test and learning set for the individual GLCM attributes—DISSIMILARITY.

**Figure 6:**
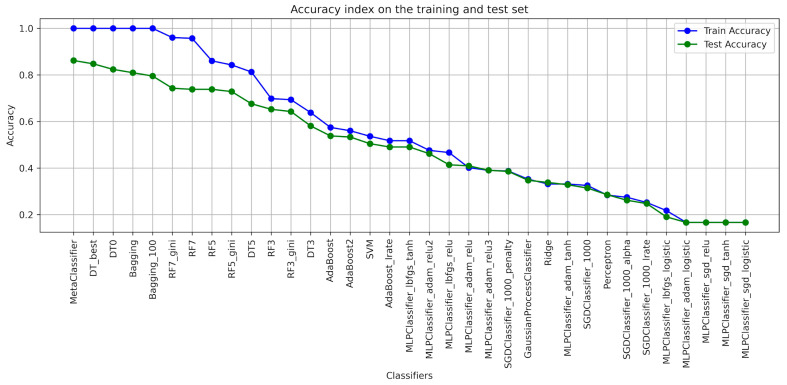
Classifier accuracy rate on the test and learning set for the individual GLCM attributes—HOMOGENEITY.

When designing the algorithms for the use of ensembles of classifiers, the architecture of each model was determined (Table 1), with the help of which the classification of blackcurrant powders with different types of carriers was applied. The results of the learning were presented with the quality indicators precision, recall, accuracy, and F1-score considering the criterion of image texture parameters using the GLCM (Table 2, Table 3, Table 4, Table 5, Table 6 and Table 7). As a result of the machine learning process, a learning quality criterion of more than 0.7 was established for the models. This means that out of 35 classifiers (Figure 1, Figure 2, Figure 3, Figure 4, Figure 5 and Figure 6), only single classifiers and a metaclassifier were selected that scored higher than the criterion.

The research on the recognition of different types of blackcurrant powders using different classification algorithms showed that the random forest algorithm was the most effective single classifier. In the context of the used carriers to distinguish currant powders, this algorithm achieved the best results. Coefficients of precision and recall, as well as F1-score, are key efficiency metrics of the extracted classifiers. It turned out that also for the random forest algorithm, all of these metrics were the highest. That means that this algorithm achieved the highest ability to accurately recognize currant powders. The high level of precision explains that random forest has a small number of false positives, while the high recall shows a high ability to detect most existing learning cases. It is worth noting that other algorithms also showed fairly high performance in recognizing fruit powders, including bagging and decision tree, among others. These aforementioned machine learning methods were also effective in recognizing currant powders, and this translates into also using them for various approaches in support of fruit powder analysis. In the result of machine learning for comparison, Table 2, Table 3, Table 4, Table 5, Table 6 and Table 7 for individual image texture characteristics using the GLCM matrix showed the result of the metaclassifier as well, which was equally effective in identifying currant powders. The metaclassifier achieved the highest result when identifying currant powders where the image attribute was entropy (Figure 1) and homogeneity (Figure 6) coefficients. In the case of contrast (Figure 3), correlation (Figure 4), and dissimilarity (Figure 5) attributes, RF_gini was the most effective single classifier. The last attribute in the GLCM related to the energy index (Figure 2) showed the highest recognition efficiency with the Bagging_100 model. In Table 2, Table 3, Table 4, Table 5, Table 6 and Table 7, it can be observed that the metaclassifier mostly has the highest precision rate with respect to all other single classifiers. This means that effective methods were selected at the data preprocessing stage for currant powders, appropriate machine learning algorithms were selected to obtain an effective metaclassifier, and good tuning of hyperparameters was achieved, obtaining optimal accuracy rates as a result of the learning process.

From the aspect of machine learning, confusion matrices are an important tool when evaluating the performance of classifiers. They allow a complete analysis of the prediction results on the test set and assist in the analysis of identifying the number of correct and incorrect classifications for each class, reflecting the performance of individual models [69]. Figure S1 shows the confusion matrices for all 35 algorithms. Relating to the performance quality criterion for the Entropy attribute, recognizing individual currant powders with different media unambiguously the metaclassifier along with the single bagging classifier showed 100% predictability for each of the 6 classes of currant powders. Each of the 35 cases were predicted as individual classes from 0–5 (presented in Figures S1–S6). A comparison of other single classifiers included Bagging_100, for which a hyperparameter n_estimators of 100 was added, suggesting to the algorithm that if the number of estimators was increased, the generalization ability would improve. As a result, the algorithm successfully classified classes except for class 2, which was predicted almost perfectly (32 out of 35 cases); the remaining cases were falsely recognized and assigned to class 1 and class 4. When analyzing the entropy parameter of the currant powder image for decision tree algorithms (DT0 and DT_best), classes 1, 3, and 5 were perfectly classified. However, when increasing the branching level for decision trees (DT5), the measure of learning accuracy deteriorates (Table 1). In the case of random forests, there is an inverse relationship of the obtained results of learning quality which translates into the results of prediction and the degree of recognition of cases for each class of currant powders. The greater the degree of branching (depth level) of random forests, the worse the accuracy in identifying results. When looking at other algorithms and the selection of hyperparameters when initializing models such as MLPClassifier, perceptron, and SGDClassfiier clearly failed in identifying individual classes of currant powders, falsely assigning all cases, usually for one class only. This confirms the difficulty of identifying multi-decisions as opposed to binary classification. The algorithms associated with AdaBoost, SVM, and GausianProcessClassfier attempted to identify individual classes only no longer as successfully as those presented in Table 1. Their accuracy rate was well below the quality criterion set for individual models.

In order to interpret the other confusion matrix (Figure S2) considering the second image texture attribute, i.e., energy (Table 3), it was observed that the confusion values are not as high as in the case of powder recognition using the entropy attribute (Table 1) but are significantly comparable to the homogeneity (Table 7) and contrast (Table 4) attributes. This is due to the fact that the metaclassifier acquired a precision quality index of 0.85 ± 0.01. Dissimilarity and correlation attributes in the case of the metaclassifier reached a much worse value.

Analyzing the contrast, dissimilarity, and correlation attributes, RF7_gini turned out to be the most effective algorithm. This model was tuned with two hyperparameters. The first one was used to generalize the model by determining the depth level (max-depth = 7) of random forests, and the second one was allowed to assess the quality of the model using the gini criterion as a criterion for the effectiveness of more precise recognition of currant powders. The confusion matrices (Figures S3–S5) for contrast, correlation, and dissimilarity do not assess the effectiveness of the algorithms in recognizing currant powders so perfectly. However, it is still possible to predict individual cases with some estimation error for currant powders and assign them as falsely recognized classes.

When predicting confusion matrices (Figure S6), due to the homogeneity attribute, there is similarity to the entropy attribute, which translates into recognition efficiency for these currant classes. The most effective models for these attributes were metaclassifier, bagging, and Bagging_100. Nevertheless, there is a certain relationship distinguishing these attributes. If currant powders were recognized with the random forest algorithm, it more effectively identified the entropy attribute than homogeneity. Attribute homogeneity grouping classification accuracy showed that decision trees are treated as the weakest algorithms. In contrast, the opposite was true for the entropy attribute. The worst results were obtained with random forest knowing that these are still classification results much higher than with homogeneity.

In the literature and research experiments, it can be shown that GLCM-based attributes are an effective tool for analyzing images, including food images. In a recent study, attributes including entropy were shown to be one of the key features for identifying cereal grains during a seeding trial with different operating speeds of a seed sowing machine [14]. In other research on strawberry powders, the uniqueness of attribute recognition using the GLCM was also demonstrated [36]. On the other hand, in the analysis of defects in infested rapeseed grains with the GLCM, the relationships between test classes were analyzed just as effectively [70]. When assessing the quality, condition, and color parameters of dried sweet potatoes based on images (indirectly through the GLCM matrix), it was also quite successful in assessing the relationships between different classes [71]. When confronted with operations on different food matrices using THE GLCM, attribute sensitivity was also noted, which may depend on the degree of structure of the object pattern and the distance between image pixels. It was also noted that modern techniques based on machine and deep learning were more effective in solving problems than what was achieved using traditional techniques. In this research applying blackcurrant powder recognition, it seemed important to select the method for the data used, i.e., microscopic images. The application of a metaclassifier proved to be the most effective model for identifying blackcurrant powders. The ensembles of classifiers extracting single classifiers, i.e., random forest, decision tree, or bagging have become a better solution than the existing neural modeling tools [47,55,58,63,72]. But the important thing is that the choice of method depends on the specific task at hand and the number of cases in the learning set.

## 4. Conclusions

In this research, we identified fruit powders with different types of carriers contained in the blackcurrant concentrate. Image texture discriminators based on the GLCM matrix distinguished the quality classes of blackcurrant powders.

The most effective classification models for fruit powders turned out to be random forest and metaclassifier algorithms. Using the metaclassifier architecture, it was possible to determine 100% classification accuracy for the entropy coefficient, 85% classification accuracy for the homogeneity coefficient, 83% classification accuracy for the energy and contrast coefficients, 77% classification accuracy for the dissimilarity coefficient, and 70% classification accuracy for the correlation coefficient. In comparison, the single random forest classifier achieved significantly approximate values for individual image texture coefficients. The application of image texture and machine learning algorithms proved to be an effective method for identifying currant powders. In fact, this merits further research in the direction of implementing machine learning methods evaluating image pixels for evaluating the quality of food products.

## Figures and Tables

**Table 1:** The structure of hyperparameters in tuning algorithms for ensembles of classifiers.

Machine Learning Algorithm Type	Name	Hyperparameters Used
DecisionTreeClassifier	DT5	max_depth = 5
DecisionTreeClassifier	DT3	max_depth = 3
DecisionTreeClassifier	DT_best	splitter = best
DecisionTreeClassifier	DT0	default
RandomForestClassifier	RF3_gini	max_depth = 3, criterion = gini
RandomForestClassifier	RF5_gini	max_depth = 5, criterion = gini
RandomForestClassifier	RF3	max_depth = 3, n_estimators = 1000
RandomForestClassifier	RF5	max_depth = 5, n_estimators = 1000
RandomForestClassifier	RF7_gini	max_depth = 7, criterion = gini
RandomForestClassifier	RF7	max_depth = 7, n_estimators = 1000
RandomForestClassifier	RF	default
AdaBoostClassifier	AdaBoost	default
AdaBoostClassifier	AdaBoost2	n_estimators = 100
AdaBoostClassifier	AdaBoost_lrate	learning_rate = 0.95
BaggingClassifier	Bagging_100	n_estimators = 100
BaggingClassifier	Bagging	default
Perceptron	Perceptron	tol = 1 × 10^−^³, random_state = 0
SGDClassifier	SGDClassifier_1000	max_iter = 1000
SGDClassifier	SGDClassifier_1000_alpha	max_iter = 1000, alpha = 0.0001
SGDClassifier	SGDClassifier_1000_lrate	max_iter = 1000, learning_rate = “optimal”
SGDClassifier	SGDClassifier_1000_penalty	max_iter = 500, penalty = “elasticnet”
RidgeClassifier	Ridge	default
MLPClassifier	MLPClassifier_adam_relu	hidden_layer_sizes = (10,10,10), activation = “relu”, solver = “adam”, alpha = 0.0001
MLPClassifier	MLPClassifier_adam_relu2	hidden_layer_sizes = (50,50), activation = “relu”, solver = “adam”, alpha = 0.001
MLPClassifier	MLPClassifier_adam_relu3	hidden_layer_sizes = (100), activation = “relu”, solver = “adam”, alpha = 0.01
MLPClassifier	MLPClassifier_adam_tanh	hidden_layer_sizes = (10,10,10), activation = “tanh”, solver = “adam”, alpha = 0.0001
MLPClassifier	MLPClassifier_adam_logistic	hidden_layer_sizes = (10,10,10), activation = “logistic”, solver = “adam”, alpha = 0.0001
MLPClassifier	MLPClassifier_lbfgs_tanh	hidden_layer_sizes = (10,10,10), activation = “tanh”, solver = “lbfgs”, alpha = 0.0001
MLPClassifier	MLPClassifier_lbfgs_logistic	hidden_layer_sizes = (10,10,10), activation = “logistic”, solver = “lbfgs”, alpha = 0.0001
MLPClassifier	MLPClassifier_sgd_relu	hidden_layer_sizes = (10,10,10), activation = “relu”, solver = “sgd”, alpha = 0.0001
MLPClassifier	MLPClassifier_sgd_tanh	hidden_layer_sizes = (10,10,10), activation = “tanh”, solver = “sgd”, alpha = 0.0001
MLPClassifier	MLPClassifier_sgd_logistic	hidden_layer_sizes = (10,10,10), activation = “logistic”, solver = “sgd”, alpha = 0.0001
GaussianProcessClassifier	GaussianProcessClassifier	default
SVM	SVM	default
MetaClassifier	mv_clf	LogisticRegression(solver = ‘liblinear’) + Bagging(n_estimators = 100) + RandomForest(n_estimators = 100)

**Table 2:** Results of learning classifiers on the test set for the individual attributes of GLCM—ENTROPY.

Algorithm AI	Accuracy	Precision	Recall	F1-Score
MetaClassifier	1.000000	1.000000	1.000000	1.000000
Bagging	1.000000	1.000000	1.000000	1.000000
Bagging_100	0.995238	0.995370	0.995238	0.995237
DT0	0.980952	0.981857	0.980952	0.980931
DT_best	0.971429	0.972848	0.971429	0.971247
RF7	0.961905	0.964733	0.961905	0.962100
RF7_gini	0.952381	0.954667	0.952381	0.952587
RF5	0.838095	0.838265	0.838095	0.836945
RF5_gini	0.833333	0.833940	0.833333	0.832052
DT5	0.795238	0.808251	0.795238	0.796725
RF3_gini	0.795238	0.796500	0.795238	0.792287
RF3	0.790476	0.792671	0.790476	0.785634

**Table 3:** Results of learning classifiers on the test set for the individual attributes of GLCM—ENERGY.

Algorithm AI	Accuracy	Precision	Recall	F1-Score
Bagging_100	0.861905	0.861827	0.861905	0.859932
Bagging	0.833333	0.834786	0.833333	0.831152
MetaClassifier	0.828571	0.841302	0.828571	0.821918
DT0	0.809524	0.810828	0.809524	0.804482
DT_best	0.785714	0.781880	0.785714	0.780262
RF7	0.761905	0.790051	0.761905	0.767754
RF7_gini	0.738095	0.765895	0.738095	0.744318
RF5_gini	0.714286	0.747303	0.714286	0.719173
RF5	0.714286	0.752105	0.714286	0.719171
DT5	0.704762	0.730829	0.704762	0.710180

**Table 4:** Results of learning classifiers on the test set for the individual attributes of GLCM—CONTRAST.

Algorithm AI	Accuracy	Precision	Recall	F1-Score
RF7_gini	0.838095	0.842470	0.838095	0.833853
RF7	0.838095	0.840120	0.838095	0.835545
DT0	0.833333	0.850281	0.833333	0.832710
Bagging_100	0.828571	0.850940	0.828571	0.825153
Bagging	0.828571	0.845043	0.828571	0.824691
MetaClassifier	0.823810	0.868227	0.823810	0.821643
RF5	0.809524	0.814468	0.809524	0.808384
RF5_gini	0.800000	0.804490	0.800000	0.799849
DT_best	0.795238	0.796844	0.795238	0.787197
RF3	0.766667	0.764466	0.766667	0.764178
RF3_gini	0.761905	0.765107	0.761905	0.762077
DT5	0.747619	0.780004	0.747619	0.745635

**Table 5:** Results of learning classifiers on the test set for the individual attributes of GLCM—COORELATION.

Algorithm AI	Accuracy	Precision	Recall	F1-Score
RF7_gini	0.719048	0.732618	0.719048	0.718729
RF7	0.704762	0.728576	0.704762	0.703350
MetaClassifier	0.700000	0.733219	0.700000	0.673960

**Table 6:** Results of learning classifiers on the test set for the individual attributes of GLCM—DISSIMILARITY.

Algorithm AI	Accuracy	Precision	Recall	F1-Score
RF7_gini	0.776190	0.777937	0.776190	0.774404
RF7	0.776190	0.772900	0.776190	0.773427
MetaClassifier	0.771429	0.797977	0.771429	0.770884
RF5	0.757143	0.747801	0.757143	0.747383
RF5_gini	0.747619	0.734150	0.747619	0.732964
Bagging_100	0.742857	0.762326	0.742857	0.745364
Bagging	0.728571	0.745875	0.728571	0.725625

**Table 7:** Results of learning classifiers on the test set for the individual attributes of GLCM—HOMOGENEITY.

Algorithm AI	Accuracy	Precision	Recall	F1-Score
MetaClassifier	0.852381	0.855450	0.852381	0.847784
Bagging	0.838095	0.840229	0.838095	0.836121
Bagging_100	0.833333	0.835938	0.833333	0.831435
RF7_gini	0.814286	0.814968	0.814286	0.811809
RF7	0.809524	0.813027	0.809524	0.805567
RF5	0.747619	0.760118	0.747619	0.742130
RF5_gini	0.742857	0.752248	0.742857	0.736238
DT_best	0.733333	0.736091	0.733333	0.725180
DT0	0.733333	0.735280	0.733333	0.728340

## Data Availability

The data presented in this study are openly available in [repository for Open Data—RepOD] at [https://doi.org/10.18150/J6R7UY], reference number [73].

## Supplementary Materials

The following supporting information can be downloaded at: https://www.mdpi.com/article/10.3390/foods13050697/s1. Figure S1: Confusion matrix of classifier ensembles calculated on the test set for the entropy attribute (GLCM); Figure S2: Confusion matrix of classifier ensembles calculated on the test set for the energy attribute (GLCM); Figure S3: Confusion matrix of classifier ensembles calculated on the test set for the contrast attribute (GLCM); Figure S4: Confusion matrix of classifier ensembles calculated on the test set for the correlation attribute (GLCM); Figure S5: Confusion matrix of classifier ensembles calculated on the test set for the dissimilarity attribute (GLCM); Figure S6: Confusion matrix of classifier ensembles calculated on the test set for the homogeneity attribute (GLCM).
