# Supplementary material for: Efficiency of Identification of Blackcurrant Powders Using Classifier Ensembles
Source: Foods. 2024 Feb 24;13(5):697. doi: 10.3390/foods13050697 (PMC10930423; doi:10.3390/foods13050697)
Supplement: Supplementary file 1 [file foods-13-00697-s001.zip › foods-2884455-supplementary.pdf]

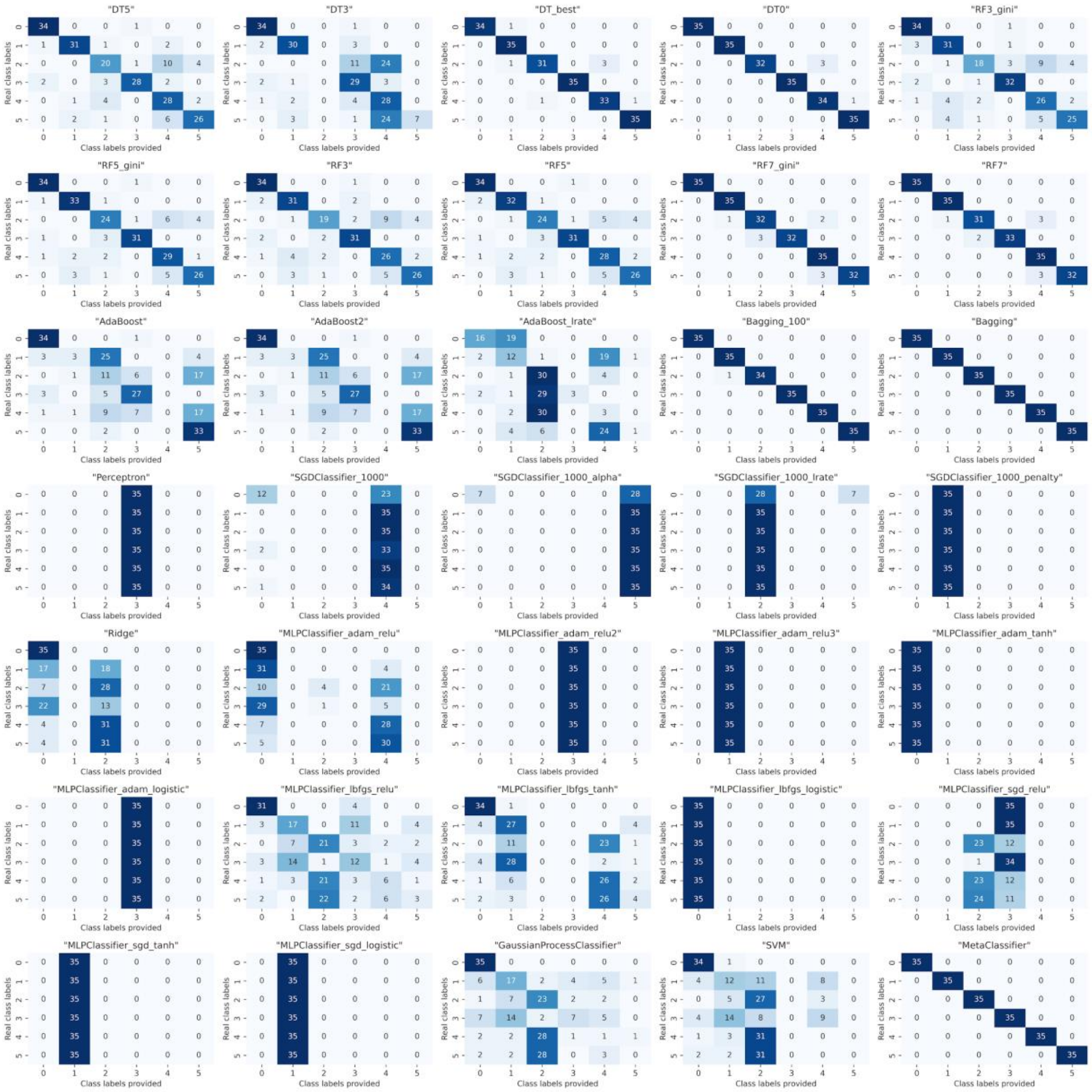

Figure S1. Confusion matrix of classifier ensembles calculated on the test set for the entropy attribute (GLCM).

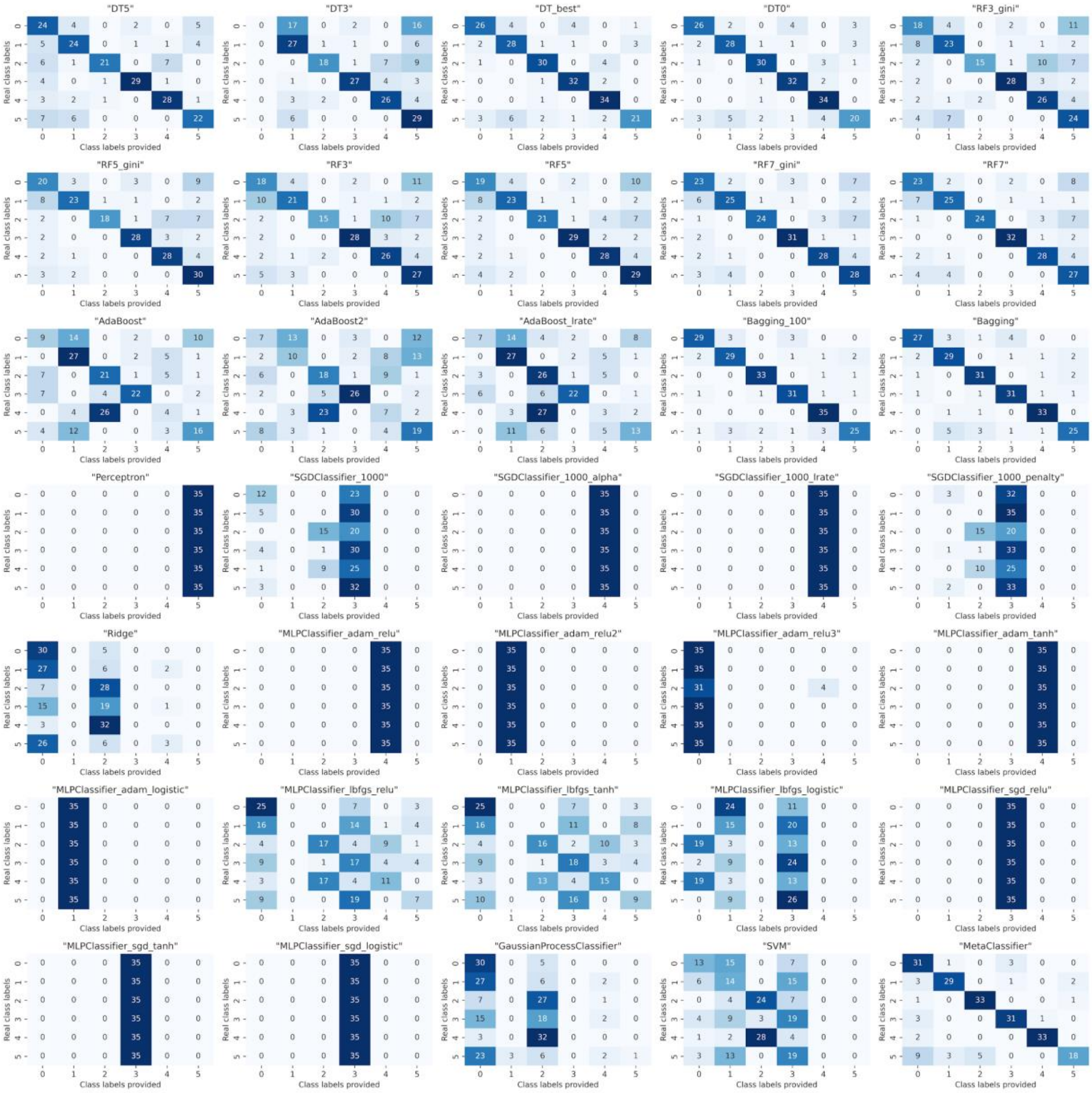

Figure S2. Confusion matrix of classifier ensembles calculated on the test set for the energy attribute (GLCM).

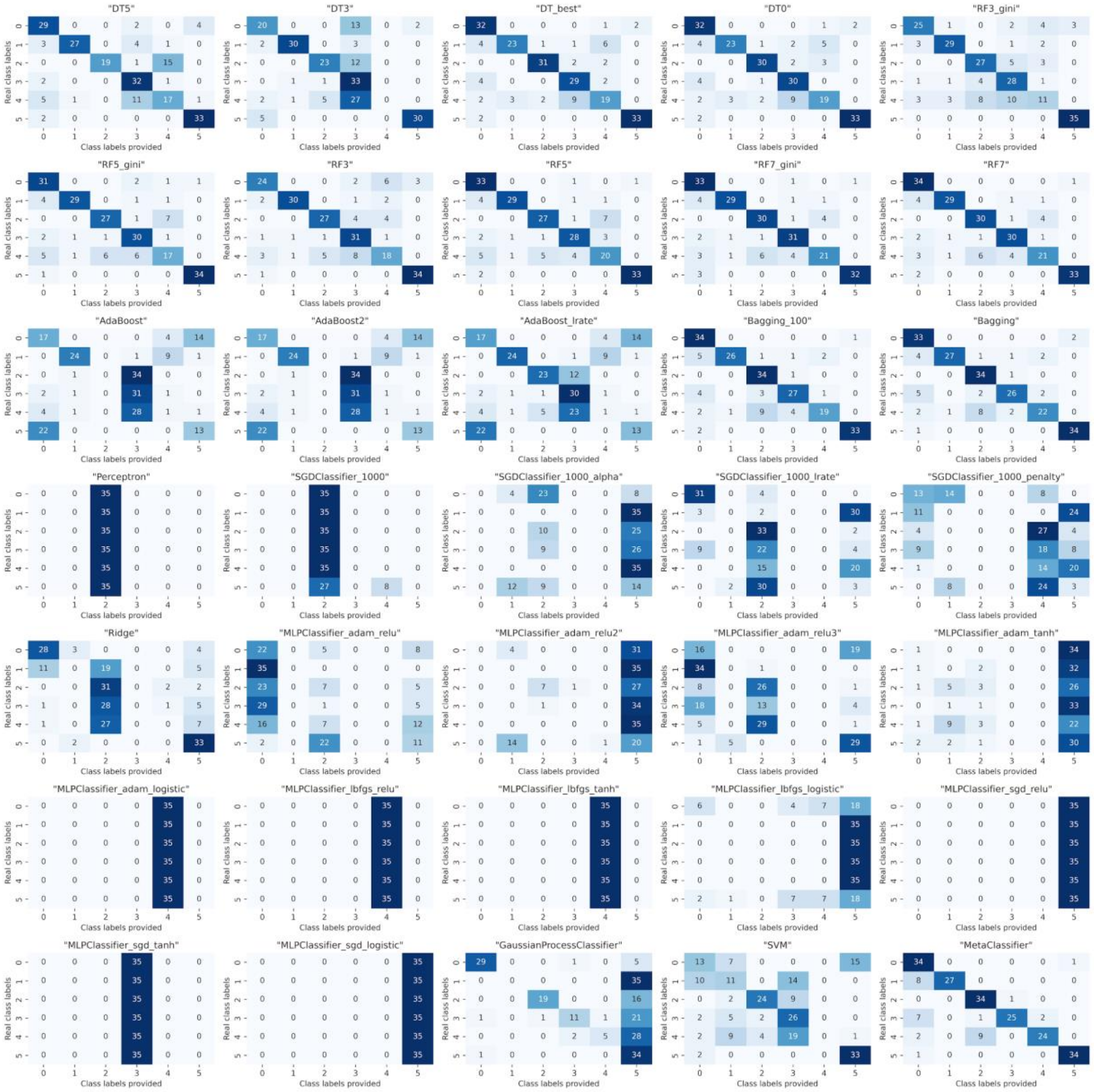

Figure S3. Confusion matrix of classifier ensembles calculated on the test set for the contrast attribute (GLCM).

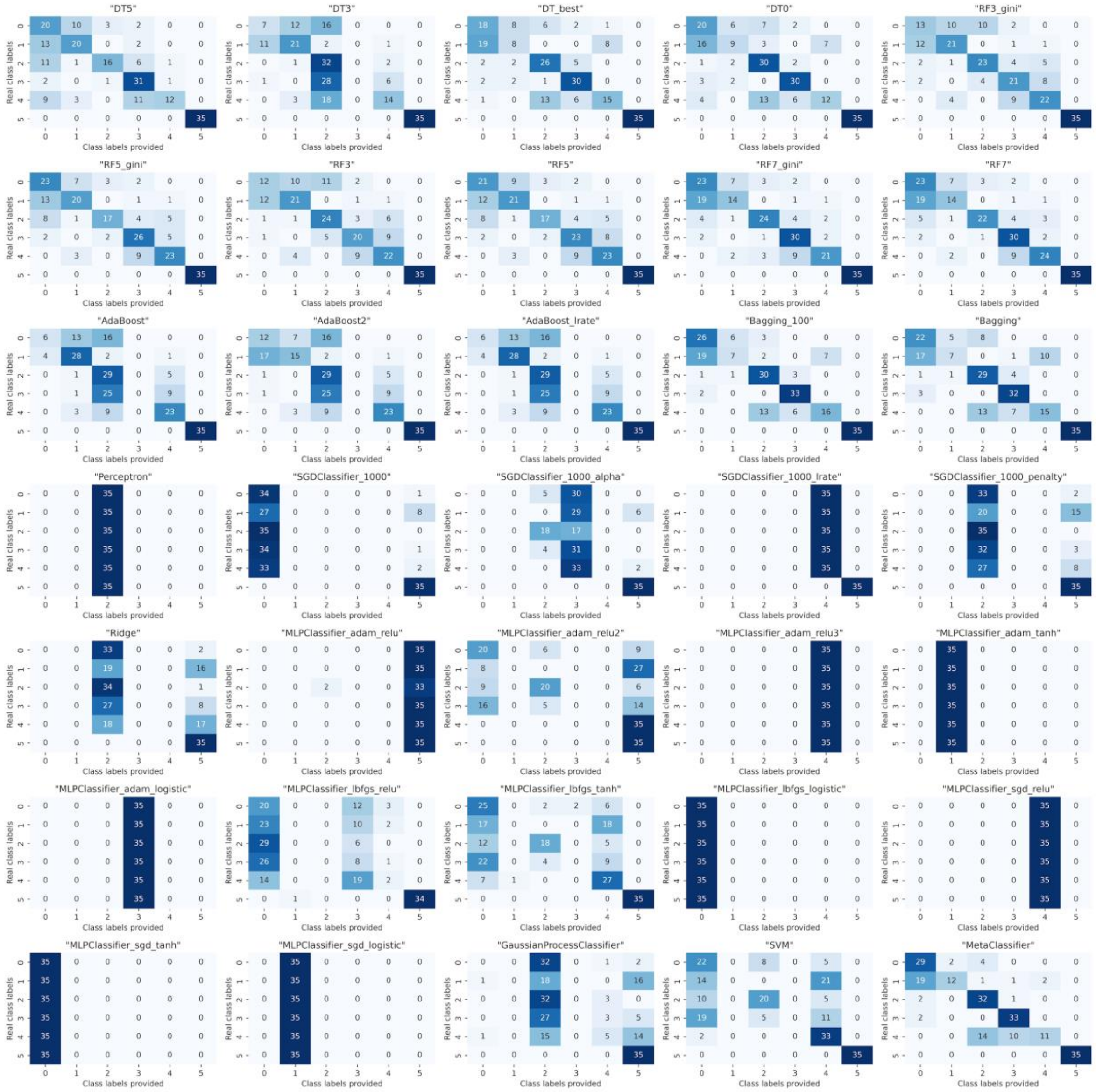

Figure S4. Confusion matrix of classifier ensembles calculated on the test set for the Correlation attribute (GLCM).

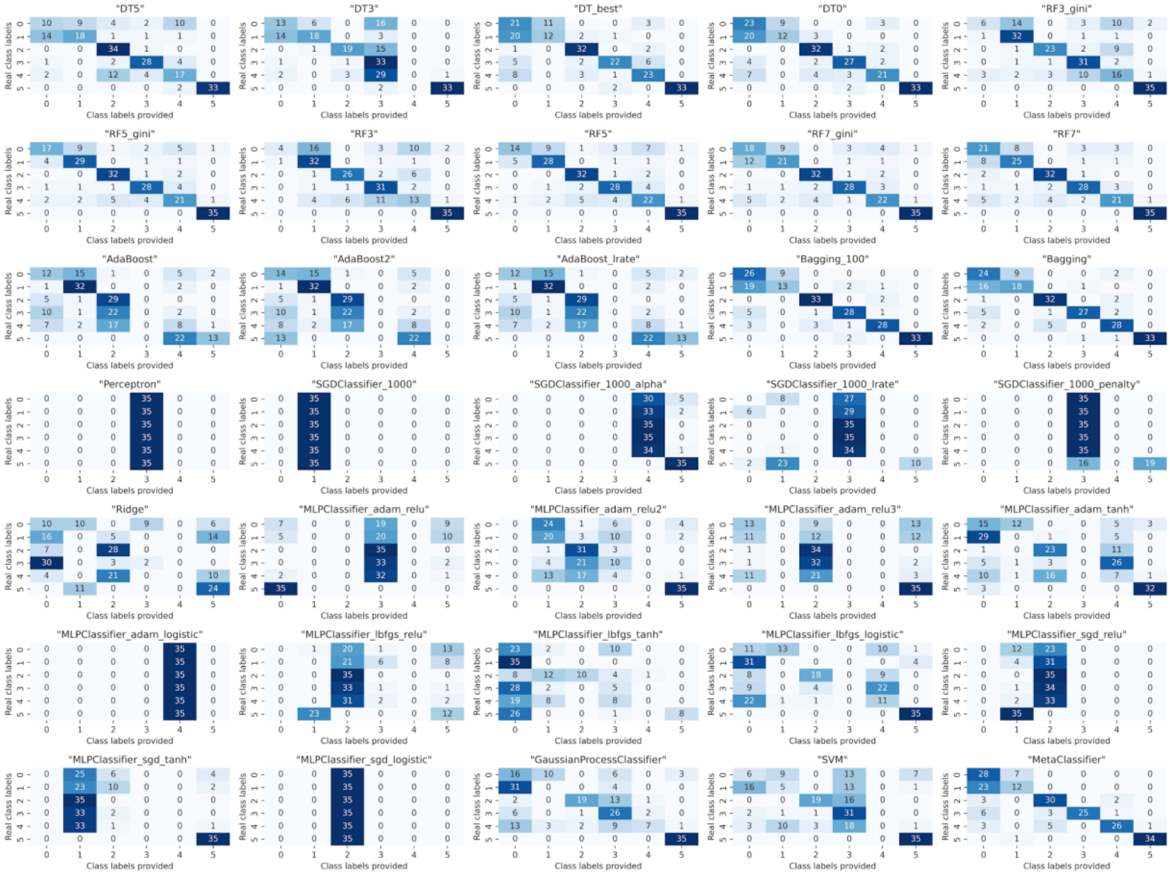

Figure S5. Confusion matrix of classifier ensembles calculated on the test set for the Dissimilarity attribute (GLCM).

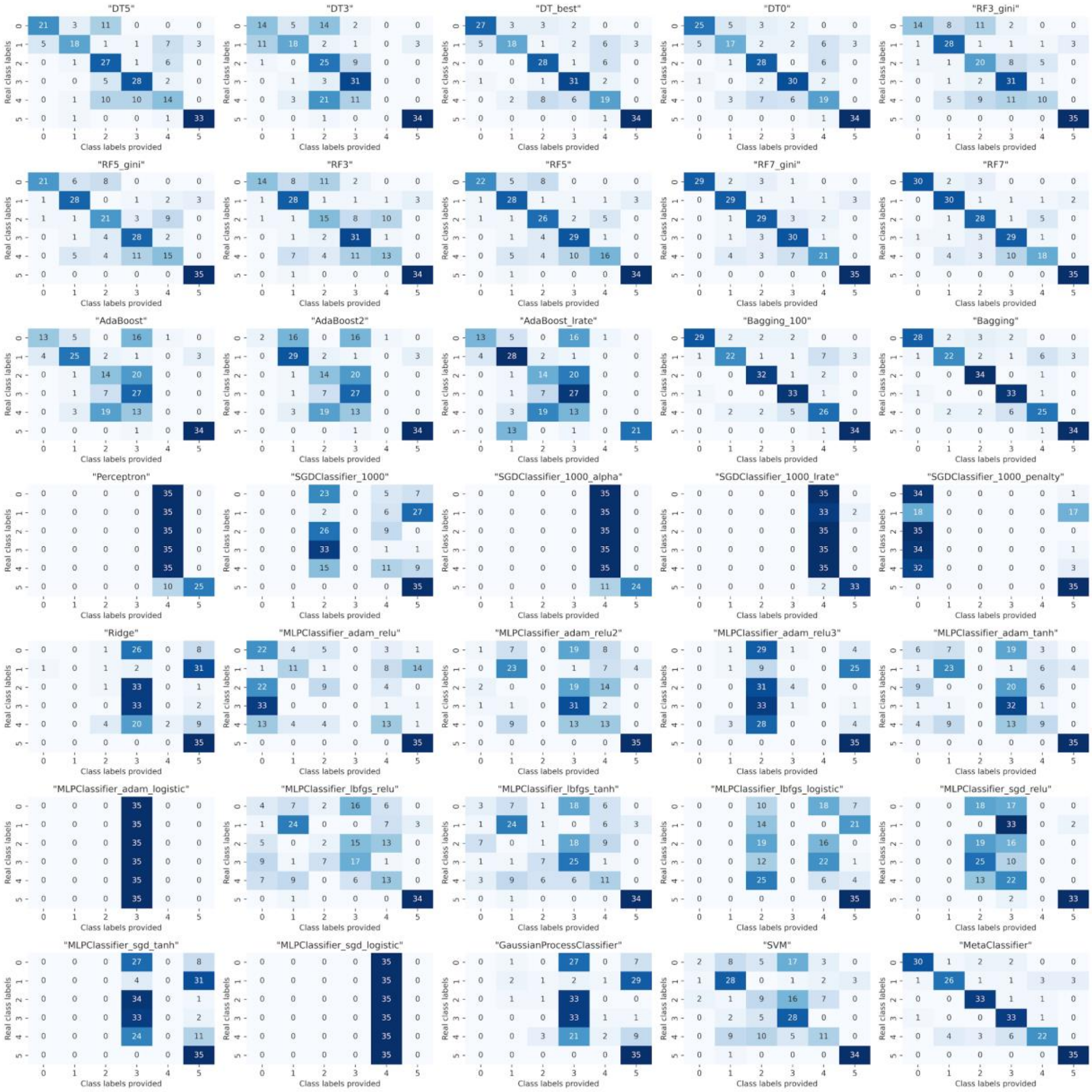

Figure S6. Confusion matrix of classifier ensembles calculated on the test set for the Homogeneity attribute (GLCM).
